# Challenges and Opportunities in Collecting and Modeling Ambulatory Electrodermal Activity Data

**DOI:** 10.2196/17106

**Published:** 2019-11-19

**Authors:** Donna L Coffman, Xizhen Cai, Runze Li, Noelle R Leonard

**Affiliations:** 1 Department of Epidemiology and Biostatistics, Temple University, Philadelphia, PA, United States; 2 Department of Mathematics and Statistics, Williams College, Williamstown, MA, United States; 3 Department of Statistics, Pennsylvania State University, State College, PA, United States; 4 Rory Meyers College of Nursing, New York University, New York City, NY, United States

**Keywords:** electrodermal activity, functional data analysis, ambulatory stress assessment

## Abstract

**Background:**

Ambulatory assessment of electrodermal activity (EDA) is an emerging technique for capturing individuals’ autonomic responses to real-life events. There is currently little guidance available for processing and analyzing such data in an ambulatory setting.

**Objective:**

This study aimed to describe and implement several methods for preprocessing and constructing features for use in modeling ambulatory EDA data, particularly for measuring stress.

**Methods:**

We used data from a study examining the effects of stressful tasks on EDA of adolescent mothers (AMs). A biosensor band recorded EDA 4 times per second and was worn during an approximately 2-hour assessment that included a 10-min mother-child videotaped interaction. The initial processing included filtering noise and motion artifacts.

**Results:**

We constructed the features of the EDA data, including the number of peaks and their amplitude as well as EDA reactivity, quantified as the rate at which AMs returned to baseline EDA following an EDA peak. Although the pattern of EDA varied substantially across individuals, various features of EDA may be computed for all individuals enabling within- and between-individual analyses and comparisons.

**Conclusions:**

The algorithms we developed can be used to construct features for dry-electrode ambulatory EDA, which can be used by other researchers to study stress and anxiety.

## Introduction

### Background

Electrodermal activity (EDA), commonly known as galvanic skin response, is a measure of sympathetic nervous system activity that is used to assess physiological arousal. Measurement of and methods for examining EDA in the laboratory setting have been well described [1], but there is less guidance for ambulatory EDA. The aim of this study was to describe and illustrate several analytic methods, specifically functional data analysis (FDA) [2] and local polynomial regression with autoregressive (AR) errors [3], neither of which have previously been applied to EDA data. We also addressed the challenges of collecting ambulatory EDA, denoising the data, and detecting meaningful features common across participants. We concluded with opportunities and limitations of the future use of ambulatory EDA data.

### Electrodermal Activity

EDA has been measured in the laboratory using gel electrodes typically placed on the palm. Entire books [1] and publication standards (eg, Society for Psychophysiological Research Ad Hoc Committee on Electrodermal Measures [4]) have been written about the measurement and the use of EDA data collected in the laboratory. However, much less has been written about the measurement of EDA in ambulatory settings [5], which has recently become popular because of the availability of dry-electrode wrist-worn devices that can be used while participants go about their daily activities. These devices have dry electrodes that are placed on the ventral wrist as opposed to laboratory placement on the palm. Emotional effects on EDA can be more sensitively recorded with electrodes on the palm rather than the wrist, but obviously, electrodes on the palm would interfere with normal activities. However, dry electrodes used in ambulatory measurement have uncertain contact with the skin in comparison with gel electrodes used in laboratory-based measurements. Important issues in the measurement of ambulatory EDA are the influence of temperature (both ambient and skin) and physical activity [4]. Several wrist-worn devices have been developed for the ambulatory collection of EDA as well as temperature and three-axis accelerometry [6,7] (eg, Affectiva Q sensor) and, more recently, blood volume pulse (eg, Empatica E4). This study used the iCalm device [8], which measures temperature and accelerometry as well as EDA (the Methods section provides a detailed description about this).

All these devices record the overall skin conductance (SC), which can be decomposed into what is referred to as tonic and phasic activity components [9–11]. Tonic activity varies slowly and is also referred to as skin conductance level (SCL). Individuals have different levels of tonic activity, that is, some individuals have a higher level of tonic activity and others have a lower level of tonic activity. In contrast, phasic activity varies rapidly in response to stimuli such as stress and is also referred to as the skin conductance response (SCR). The SCR amplitude is considered to be a measure of sympathetic activity. SCR is characterized by a rapid incline to a peak and then a slower decline back to the individual’s SCL [1]. Typically, the goal is to detect these peaks and compute their features (eg, amplitude), which can serve as predictors or outcomes in statistical models. Although the features can be detected by visualizing the data and coding the peaks, the amount of data that are typically collected in ambulatory research studies makes this time consuming and impractical. Next, we introduce the study from which the data were obtained before returning to approaches for analyzing ambulatory EDA data.

### Power Source Parenting Study

Many adolescent mothers (AMs) have challenges effectively regulating emotion, which interferes with their use of positive parenting skills and places them at risk of maltreating their children. Data for this study were collected from participants in a pilot, group-randomized controlled trial of the Power Source Parenting (PSP) intervention for high-risk AMs [12,13]. The AMs were homeless and lived with their children in transitional living programs (TLPs) in a Northeast state. Each participant received a baseline and 2 postintervention follow-up assessments (at 3 and 6 months). Each assessment lasted between 1.5 and 2 hours, and the AMs wore the iCalm biosensor band that measured EDA throughout each of the 3 assessments, which included 2 potentially stressful tasks: (1) a timed Stroop task and (2) a 10-min videotaped mother-child interaction. We examined EDA at each assessment for the Stroop and video interaction tasks. We chose these 2 tasks because we expected that they induce different types of stressors (eg, Stroop task is a cognitive stressor) and they have differing degrees of structured activity. The Stroop task was more structured than the videotaped interaction task, and it more closely resembles the measurement of EDA in the laboratory (ie, AMs complete the computerized task seated alone without her child present). In contrast, the semistructured mother-child videotaped interaction does not have the constraints of carefully controlled laboratory settings but provides more ecologically valid information about maternal and child behaviors concurrent with measurement of AMs’ EDA that can be examined across participants.

In our study, different AMs had quite different features in their EDA profiles (ie, the EDA level over the time span of a single task), making it difficult to compare them directly. Some of the differences are noise, such as those from movement artifacts. Despite innovations in devices, ambulatory assessment of EDA is inherently noisier than laboratory assessment because of these movement artifacts and the dry electrodes. Thus, the first step in processing EDA data is to identify these artifacts so that these data can be discarded. The step typically involves applying a signal processing filter, such as a Hanning filter with a 1-second window [14], a zero-phase order 10 low-frequency Butterworth filter [5], or a finite impulse response (FIR) low-pass filter [15]. The application of a signal processing filter is not mandatory, as the next step, identifying valid versus invalid data, can be applied directly to the raw, unfiltered data [15]. Taylor et al [15] developed an algorithm using support vector machines (SVMs) to identify valid versus invalid data and applied it to both the raw SC data and SC data filtered using an FIR low-pass filter. SVMs have a disadvantage in that it is not transparent what rules are being implemented to classify data as valid versus invalid. In contrast, a rule-based algorithm was proposed [16] for classifying data as valid or not. For example, values of less than 0.05 μS could be excluded [5]. A disadvantage of this type of approach is that it is less flexible; however, it does not have the reproducibility problems that the Taylor et al [15] approach may have [16]. Usually, a combination of a signal processing filter and either a rule-based approach or a machine learning approach is used to identify and discard invalid data [5,15]. Given the valid data, the next step is to separate the tonic and phasic components.

Many of the previous methods for separating the components are based on EDA data collected in the laboratory in which the participant is exposed to a stimulus, such as a loud noise, at regular intervals (eg, interstimulus interval of 8 seconds). These methods include the sigmoid exponential model [11] and negative deconvolution [9]. Although these methods were developed for data collected in the laboratory, the negative deconvolution method has been applied to ambulatory data [14].

There is a question as to whether it is important to separate these components in ambulatory data because the stimuli are typically more gradual (typically referred to as nonspecific SC responses [1]) as opposed to the abrupt startle response stimuli typically used in laboratory research. Hernandez et al [14] analyzed the data without decomposing the tonic and phasic components and by decomposing them using nonnegative deconvolution and found that the decomposition provided more discriminative information. Thus, they recommended decomposing the EDA. Regardless of whether the EDA is decomposed or not, the next step is to identify features of the data, typically peaks, and compute the characteristics of these features.

**Figure 1.**
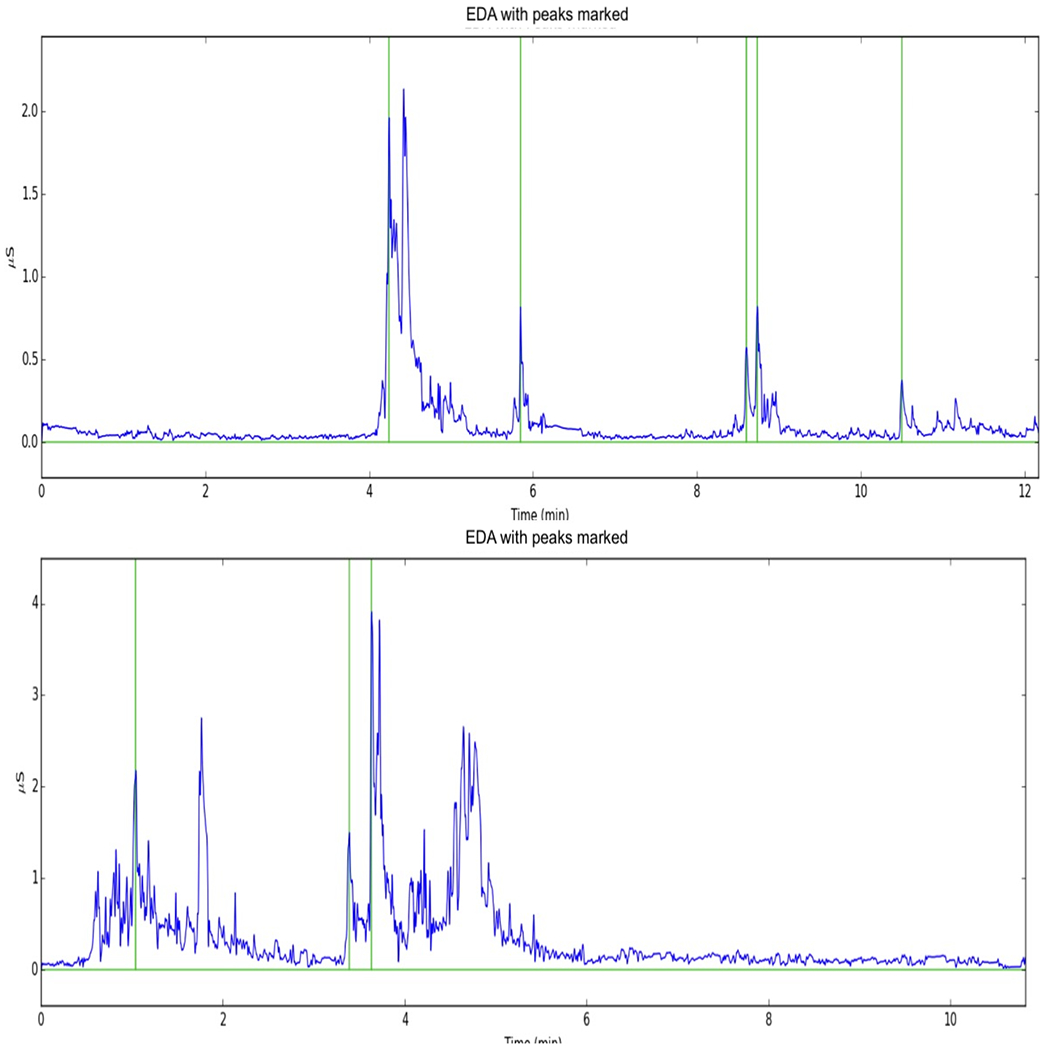
Electrodermal activity peak detection results for the free-play (first 5 min) and teaching (second 5 min) videotaped interaction tasks for one control group mother (patient ID=102) and at baseline and 3-month follow-up assessments. EDA: electrodermal activity.

One automated approach to detecting peaks and constructing features is available on an interactive website at the MIT Media Lab [15] and uses SVMs, although the focus was primarily on identifying valid versus invalid data. Kleckner et al [16] also did not focus on detecting peaks but only on identifying valid versus invalid data. Thus, there is very little guidance on detecting peaks once data have been identified as valid. We initially used the Taylor et al [15] approach and discovered that the approach did not identify many of the peaks and marked much of the data as invalid. The results of applying this approach are presented in Figure 1 for one AM as an example and are what motivated us to try to find a better approach to detecting peaks in the ambulatory setting.

In general, our approach was to (1) use the raw SC data or SC data filtered with an FIR low-pass filter, (2) identify valid versus invalid data using rules established in the literature, (3) decompose the signal into its tonic and phasic components using negative deconvolution, (4) apply a spline-based smoothing technique (eg, B-spline regression or local polynomial regression with AR errors) to the EDA profile, and (5) compute various features of the peaks identified from the smoothed curve. The latter 2 steps, particularly the spline-based approaches, are the unique contributions of this study. Comparisons between different groups of individuals can then be performed on the peak features, if desired.

## Methods

### iCalm Sensor Band

The iCalm applies a constant voltage to the skin through 2 silver/silver chloride metal electrodes and measures the resulting current [8,13]. A transconductance amplifier is used to convert the current to a voltage, which is sampled at a rate of 4 Hz using a 12-bit analog-to-digital converter. Three-axis accelerometry and temperature were sampled at the same rate. The iCalm has been validated against laboratory measures of EDA [6]. The iCalm connects via Bluetooth to Android-based mobile phones.

### Participants

The data were obtained from the aforementioned PSP intervention study of 71 mothers. The EDA values for some mothers were very low, which was primarily because of a lack of contact between the skin and the electrodes. The general guidance in the literature is to discard data in which the EDA is approximately 0 [16], as it indicates that the electrodes were not in good contact with the skin. Details regarding these invalid data are given in section Data Preprocessing. Finally, there were 5 AMs who did not return at any of the follow-up assessments and several AMs who had data at one but not both of the follow-up assessment periods; thus, we used 6-month follow-up data if an AM did not have data at the 3-month follow-up. The final sample size numbers are given following discussion of the identification of valid versus invalid data.

### Procedures

The AMs were interviewed at the TLP in a private room by trained interviewers. The interviews included a survey questionnaire examining demographic and background characteristics; questions regarding mental health, parenting, and risk-taking behaviors; as well as activities described in the Measures section. AMs wore the iCalm band on their right wrist throughout the duration of each interview. Following the interview, mothers in the intervention TLPs received the PSP intervention, whereas the control TLPs received standard care [12].

### Measures

Each assessment included the computer-delivered survey, a variety of computerized tasks including the Stroop task, and the 10-min videotaped interaction of mothers with their child. In this analysis, we focused on the Stroop task and the videotaped interaction task. The Stroop task involved reading the written color names independent of the color name that is written as quickly as possible in 1 min. For the videotaped interaction task, the mothers were asked to play with their child as they usually do for the first 5 min, which we will refer to as free play. For the second 5 min of the interaction task, the mothers were asked to teach their child a concept that was slightly above their developmental level. This latter task, which we will refer to as teaching, was intended to be slightly frustrating for the mother and the child. In addition, a physical task (going up and down stairs for approximately 5 min) was only performed at the baseline assessment and only for the intervention group AMs. We examined these data as a comparison task in which there is a lot of movement.

### Data Analysis

The data analysis for analyzing and comparing the EDA profiles entails several steps described earlier, including preprocessing, smoothing, identifying peaks, computing features of the peaks, and, finally, statistical modeling. In this section, we present the details of these steps with regard to our data. The R code for implementing the analysis is provided in Multimedia Appendix 1. To date, software for identifying invalid data, decomposing SC, detecting peaks, and constructing features has been written in either MATLAB or Python. To our knowledge, there are no R packages for handling these tasks.

#### Data Preprocessing

*Data preprocessing* involved computing the elapsed time since the beginning of the Stroop and video interaction tasks for each mother at the baseline and follow-up assessments so that both assessments and all AMs would be on the same time scale. Next, we constructed plots of EDA for each mother by task to visualize motion artifacts and loose band connections. For the filtered data, we generated an FIR low-pass filter using the function *firl* in the R package *signal* and chose the number of coefficients as 16 (ie, the sample rate×4). We considered EDA values that were approximately 0 as invalid, as these usually indicate that the band had a loose connection and did not accurately record EDA [5]. These data were removed from both the filtered and raw SC data. We also examined plots of the EDA during the physical task to ensure that, as expected, EDA increased with physical activity and that the band had a good connection. Finally, each mother at each of the baseline and follow-up assessments had differing ranges of EDA values (eg, refer to the y-axis in Figure 1). Hence, in accordance with standard practice [14], we normalized EDA values by the range and distance from the maximum value:

$$\text{normalized}.\text{EDA}=1-\frac{\max.\text{EDA-EDA}.\text{total}}{\max.\text{EDA-min}.\text{EDA}}$$

where the one minus is to correct the sign of the normalized EDA values to be the same as the original EDA values. Next, we used 2 methods for *data smoothing:* FDA with B-splines and local polynomial regression for data with AR errors.

#### Functional Data Analysis

FDA considers the EDA profile as a single entity rather than as a collection of data points. Missing data or unequally spaced measurements are smoothed over. The target function is estimated by a linear combination of basis functions, specifically spline functions (hence the name B-splines) of a specific polynomial order *m*. The entire range of the target function is divided into subintervals at breakpoints called knots. Splines are then fitted in each interval and are adjusted to join the knots to estimate the underlying smoothed function. We also added a penalty on the roughness of the fitted curve, with the tuning parameter chosen by the generalized cross-validation (GCV) index. In addition, the number of knots was chosen by the method proposed by Ruppert [17]. We implemented this approach using the *fda* R package to smooth the data and create functional profiles. Specifically, we used the function *create.bspline.basis* to generate B-spline basis functions and then used the functions *fdPar* and *smooth.basis* to produce the fitted functional form as well as the corresponding value for the GCV index. The first 2 derivatives of the estimated function were calculated and saved for future use. The estimated smoothed curve captures the general features of the normalized EDA profile. The results could change significantly for the choice of location of knots and the number of knots. We chose the knots to be equally spaced on the time span, but it is not necessary that they be equally spaced [18]. If they are not equally spaced, then the spacing may need to be customized for each individual. For example, knots could be placed more closely together when the EDA profile is changing significantly and placed further apart when the EDA profile does not change as much (ie, when the EDA profile is flat).

#### Local Polynomial Regression

*Local polynomial regression with AR errors* is another technique to obtain a smooth estimate of the EDA profile. We applied a version of local linear regression for data with AR errors [3] because the EDA data in a profile is longitudinal and from the same individual (hence correlated). This approach assumes that the response variable is the sum of a smoothed unknown function of time and an error term, where the error term follows an AR model with d terms:

$$y_{t}=m\left(t\right)+\varepsilon_{t},\text{with}\varepsilon_{t}=\beta_{1}\varepsilon_{t-1}+\beta_{2}\varepsilon_{t-2}+\cdots +\beta_{d}\varepsilon_{t-d}+\eta_{t}.$$

For a fixed *d*, the estimation procedure starts by obtaining an initial estimate ${\hat{m}}_{I}\left(t\right)$ by assuming working independence among observations and then calculating the corresponding residuals $\hat{\in }\left(t\right)$ The initial estimate ${\hat{m}}_{I}\left(t\right)$ is calculated by local linear regression with the plug-in bandwidth selector. The problem can be converted to a partial linear problem as follows:

$$y_{t}=m\left(t\right)+\beta_{1}{\hat{\varepsilon }}_{t-1}+\beta_{2}{\hat{\varepsilon }}_{t-2}+\cdots +\beta_{d}{\hat{\varepsilon }}_{t-d}+\eta_{t}$$

and m(⋅) and $\beta^{\prime }s$ are estimated by profile least squares. More specifically, we first estimate $\beta^{\prime }s$ by the so-called *difference-based method* [19] and then derive a local linear smoother on the difference:

$$y_{t}^{*}=y_{t}-\left({\hat{\beta }}_{1}^{dbe}{\hat{\varepsilon }}_{t-1}+{\hat{\beta }}_{2}^{dbe}{\hat{\varepsilon }}_{t-2}+\cdots +{\hat{\beta }}_{d}^{dbe}{\hat{\varepsilon }}_{t-d}\right)$$

as an estimate of the final m(⋅). The number of AR terms, *d*, is selected by a penalization method, with the tuning parameter selected by the Bayesian information criterion [20]. The local linear approach may not work well in cases where the fitted curve is lower than the actual EDA profile or is influenced by some outliers with extreme values. The local linear regression may also over smooth the fitted curves and therefore miss capturing peaks in the EDA profiles. In these cases, local quadratic regression or local cubic regression may yield better results. We applied both the local quadratic and local cubic regressions, but the fit was similar to the local linear regression; hence, we present only the local linear regression results in the Results section. Other literature [15] on analyzing EDA data suggests using a low-pass filter to denoise data first. Thus, we also tried the combination of an FIR low-pass filter and local linear regression as well as using local linear regression alone.

#### Identifying Peaks

Using the smoothed EDA profiles, our interest lies in identifying features related to significant local maxima (ie, peaks). Correct peaks should increase quickly and then decrease more slowly, with large enough rise and drop [1]. To identify peaks, we used the first and second derivatives saved earlier during the spline smoothing estimation. The local maxima are locations where the first derivative is 0 and the second derivative is negative. We first found all local maxima and then filtered them by the magnitude of the rises/drops. Specifically, for each local maximum, we identified the nearest local minimum on each side and then calculated the corresponding drop. If both drops are larger than a preset threshold, the local maximum is identified as a peak. The threshold was set to 0.1 [1]. There could be an incomplete peak at the right limit of the EDA profile, which we did not identify as a useful peak even if its drop satisfies the 0.1 criterion.

#### Computing Features of the Identified Peaks

To make a comparison between different individuals, we computed the characteristics or features of the identified peaks, including the number of identified peaks, time to the first peak, time to the highest peak, and maximum value (ie, amplitude) at the first peak. Initially, we also considered the area under the entire smoothed curve. However, the actual observed time span for each individual was not exactly the same, although the actual task was designed to last the same amount of time, making the area under the curve (AUC) not very comparable across individuals for a given task. Nevertheless, for data in which the time span is the same for each individual, AUC may be a meaningful feature. For each task, we calculated the abovementioned peak features for each AMs’ EDA profile. The R code for identifying and computing the features of the peaks is provided in Multimedia Appendix 1.

The final step is statistical modeling using the features of EDA peaks. This step depends, of course, on whether there are groups to be compared and repeated features on the same individual (eg, baseline and postintervention). Thus, any statistical model could potentially be used depending on the characteristics of a particular study.

## Results

We began by examining plots of the EDA for each mother by task. Graphical display of the data showed EDA varied both between individuals and within individuals during both tasks. Owing to space, we did not present figures for all the AMs. Instead, we focused on one control group AM (patient ID [PID] 102) and one intervention group AM (PID 202).

**Figure 2.**
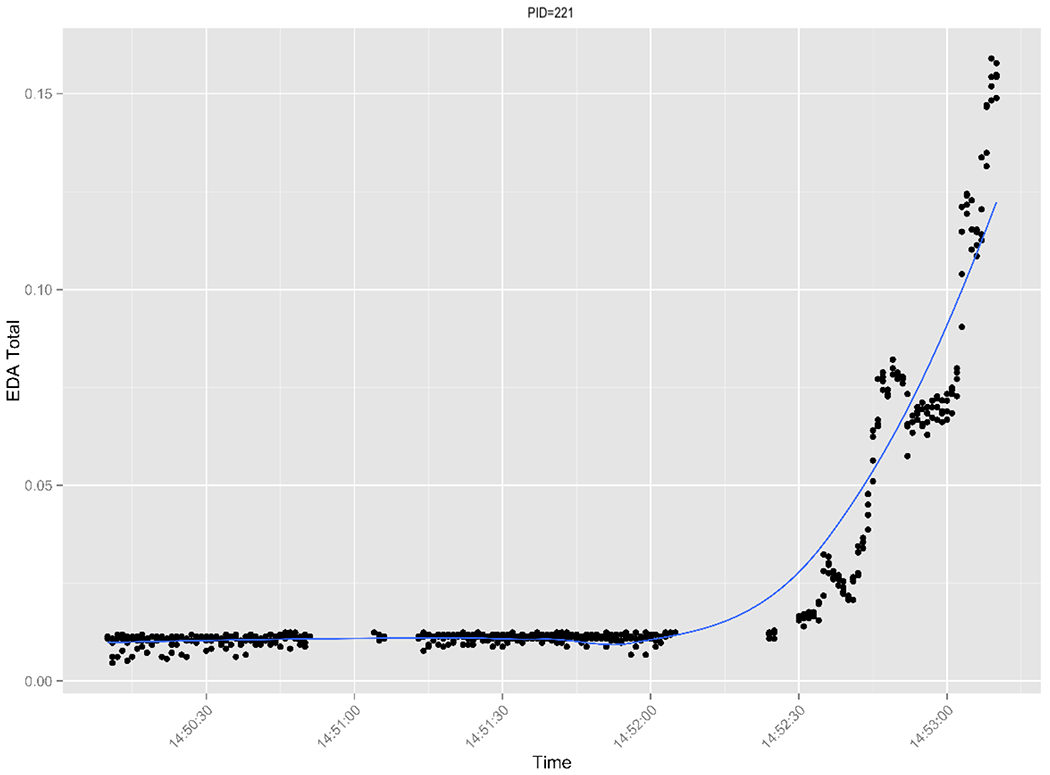
Electrodermal activity for physical task at baseline for one intervention group mother. A loess curve has been fit to the data. EDA: electrodermal activity; PID: patient ID.

We also examined plots of the EDA data for the physical task for the intervention AMs at baseline. The examination of these plots revealed that the AMs’ EDA increased, as it should (Figure 2 shows an example plot of one mother) during the physical task.

### Local Polynomial Regression

**Figure 3.**
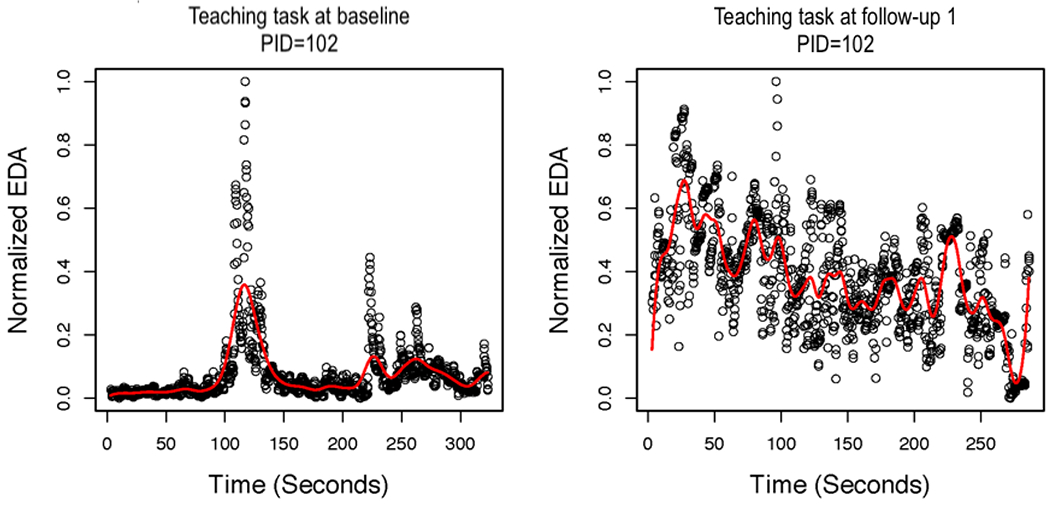
Local linear regression with autoregressive errors for the teaching task at baseline and 3-month follow-up assessments for one mother (patient ID=102). EDA: electrodermal activity; PID: patient ID.

**Figure 4.**
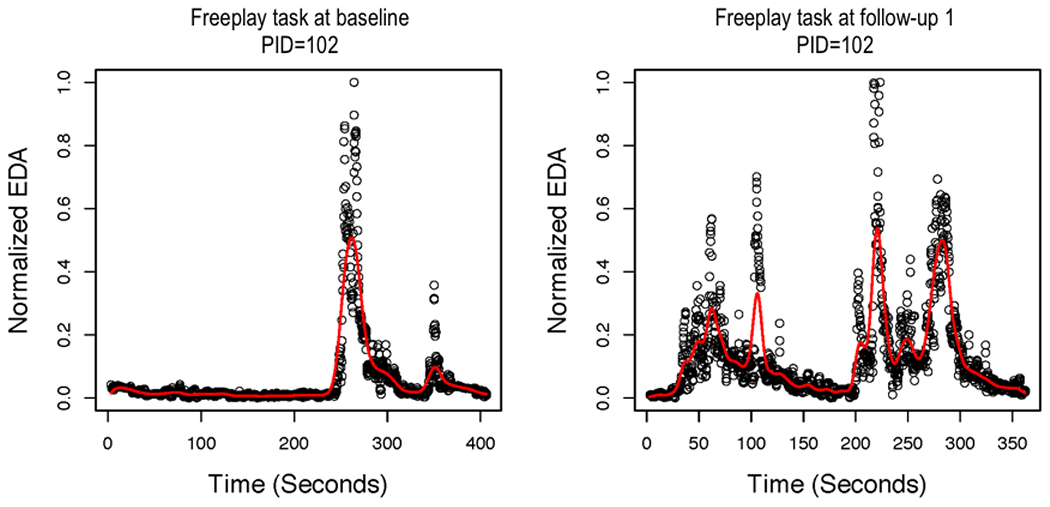
Local linear regression with autoregressive errors for the free-play task at baseline and 3-month follow-up assessments for one mother. EDA: electrodermal activity; PID: patient ID.

**Figure 5.**
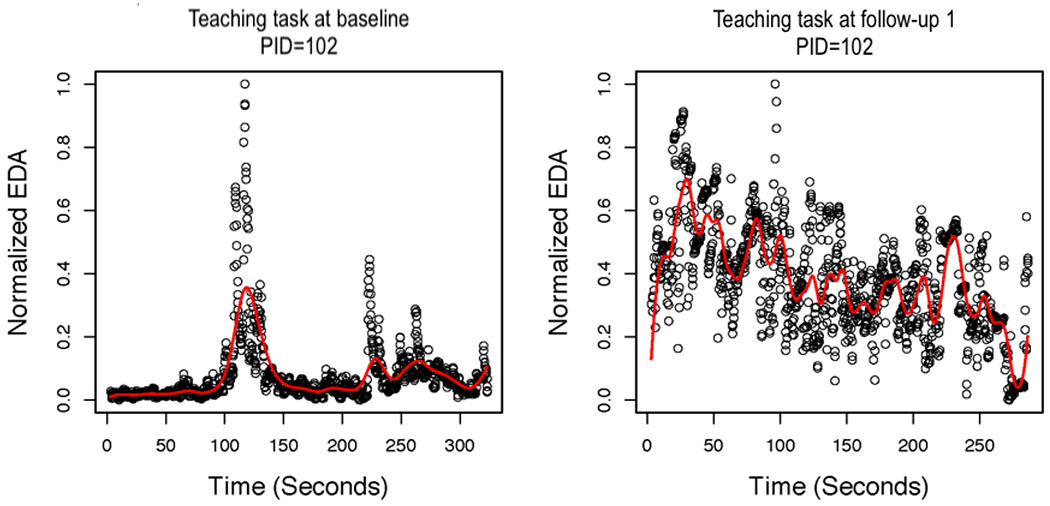
Local linear regression with autoregressive errors for the teaching task at baseline and 3-month follow-up assessments for one mother after applying a low-pass filter. EDA: electrodermal activity; PID: patient ID.

**Figure 6.**
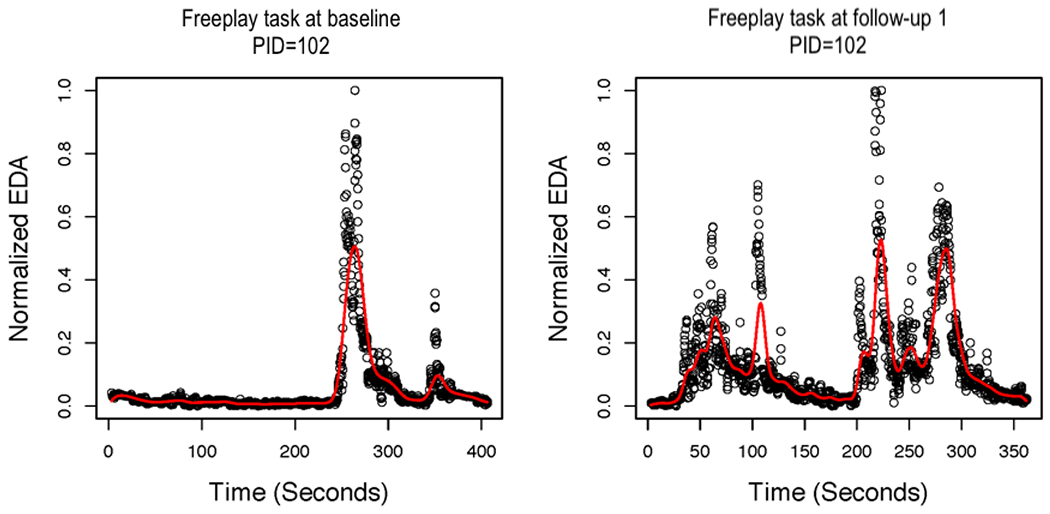
Local linear regression with autoregressive errors for the free-play task at baseline and 3-month follow-up assessments for one mother after applying a low-pass filter. EDA: electrodermal activity; PID: patient ID.

Next, we present the results of the local linear regression with AR errors. Figure 3 shows the smoothed function overlaying the normalized EDA values for the baseline and 3-month follow-up assessments for the teaching task for one control group AM. Figure 4 presents the smoothed function overlaying the normalized EDA values for baseline and 3-month follow-up assessments for the free-play task for the same AM, as presented in Figure 3. In the case of this AM, the smoothed function does not fit the actual peaks in the data very well. Specifically, the smoothed peaks are dampened in comparison with the actual peaks, which creates a problem for identifying peaks because they are defined by their increase/decrease. Even if peaks are identified, some features of the peaks (eg, amplitude) would be dampened in comparison with the actual data. We also tried using an FIR low-pass filter before smoothing with local linear regression for AR errors. However, this did not substantially improve the fit of the estimated functions to the actual data. Figure 5 shows the smoothed function overlaying the normalized EDA values after applying the FIR low-pass filter for the baseline and 3-month follow-up assessments for the teaching task for the same AM, as presented in Figure 3. The free-play task is presented in Figure 6. It is evident that the FIR low-pass filter did not change the estimated EDA profiles substantially from the raw SC data. We next attempted smoothing using B-splines to estimate the functional EDA profiles.

### Functional Data Analysis

**Figure 7.**
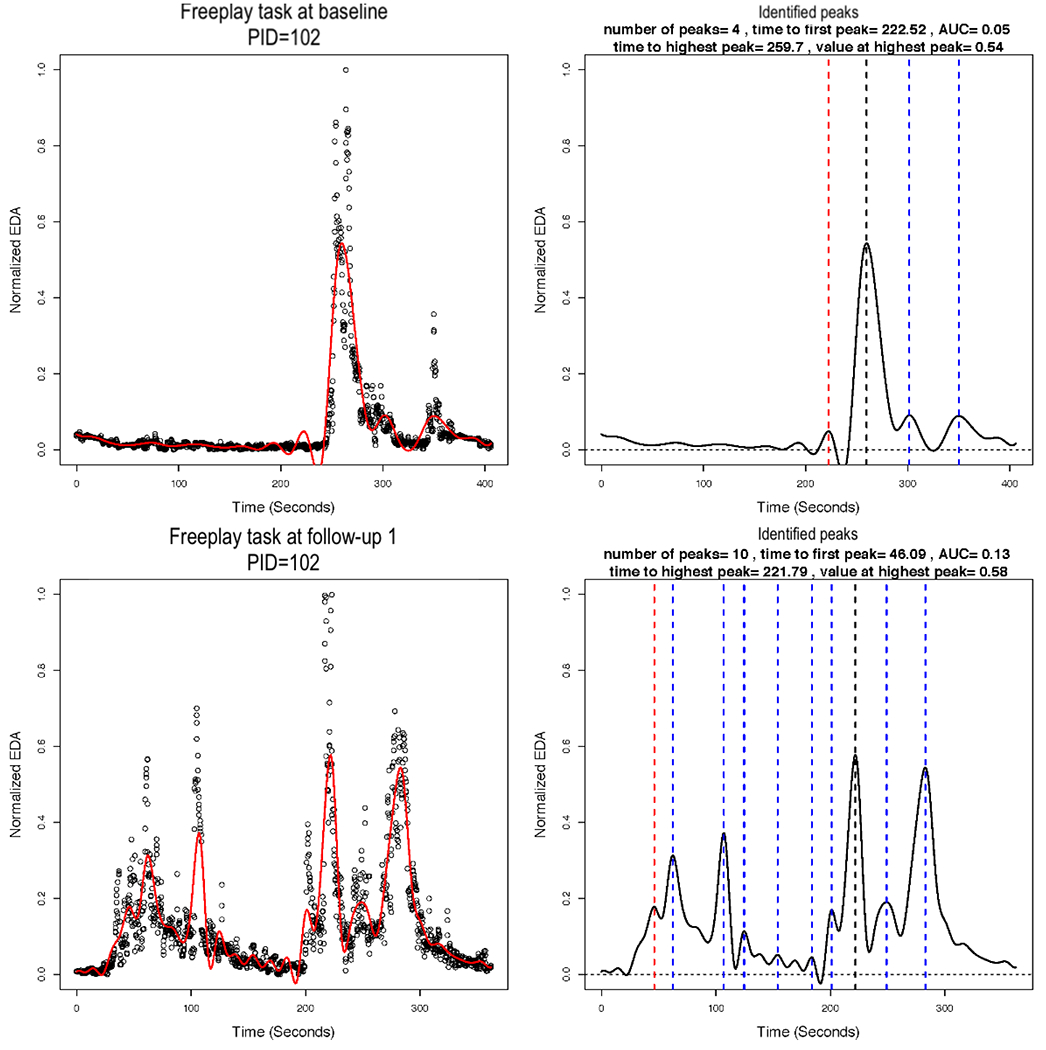
Electrodermal activity (EDA) for the free-play task at baseline and 3-month follow-up assessments for one mother (patient ID=102). The left column shows the estimated B-spline smoothed function overlaying the normalized EDA values. The right column shows the identified peaks and the values for peak features. The red dashed vertical lines demarcate the first peak, the black dashed vertical lines demarcate the highest peak, and the blue dashed vertical lines demarcate all other identified peaks. AUC: area under the curve; EDA: electrodermal activity; PID: patient ID.

**Figure 8.**
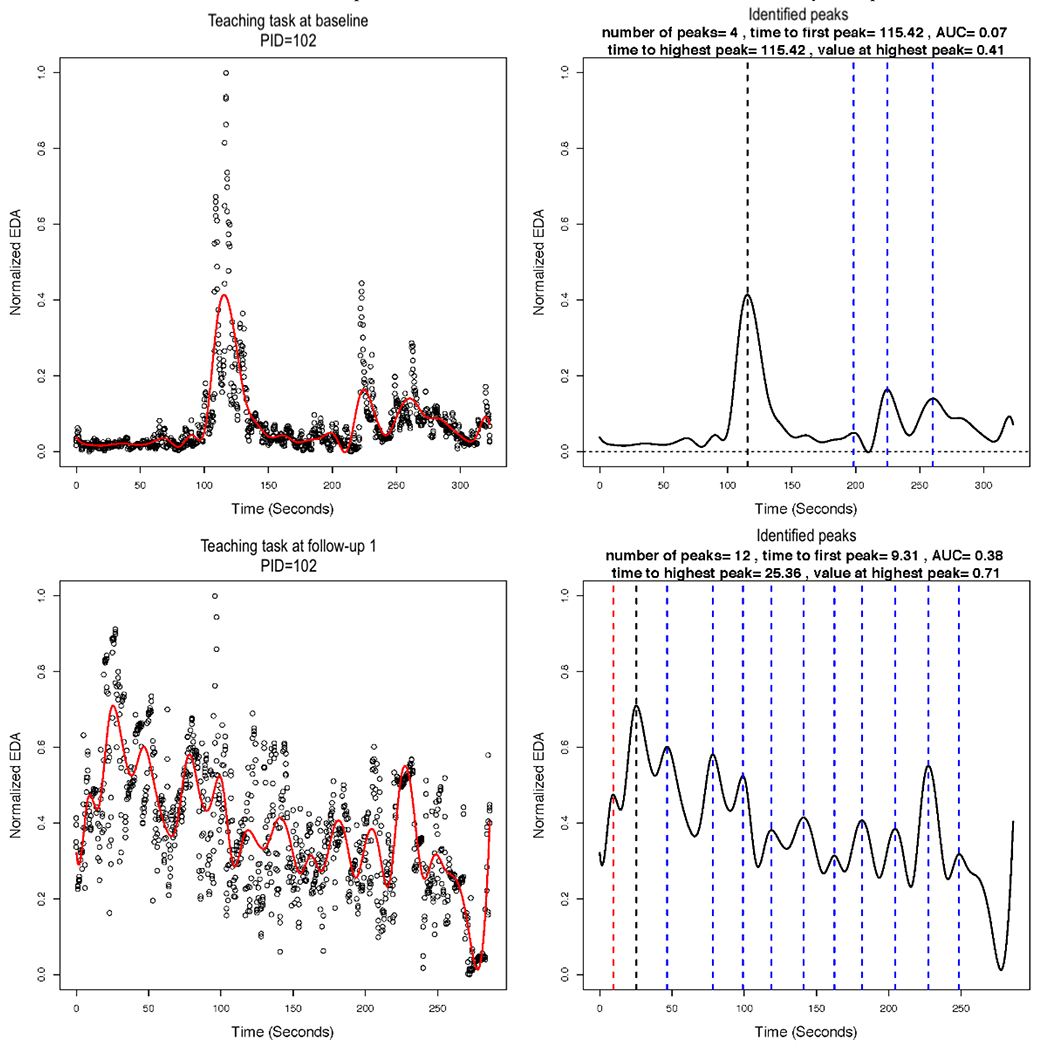
Electrodermal activity (EDA) for the teaching task at baseline and 3-month follow-up assessments for one mother (patient ID=102). The left column shows the estimated B-spline smoothed function overlaying the normalized EDA values. The right column shows the identified peaks and the values for peak features. The red dashed vertical lines demarcate the first peak, the black dashed vertical lines demarcate the highest peak, and the blue dashed vertical lines demarcate all other identified peaks. AUC: area under the curve; EDA: electrodermal activity; PID: patient ID.

**Figure 9.**
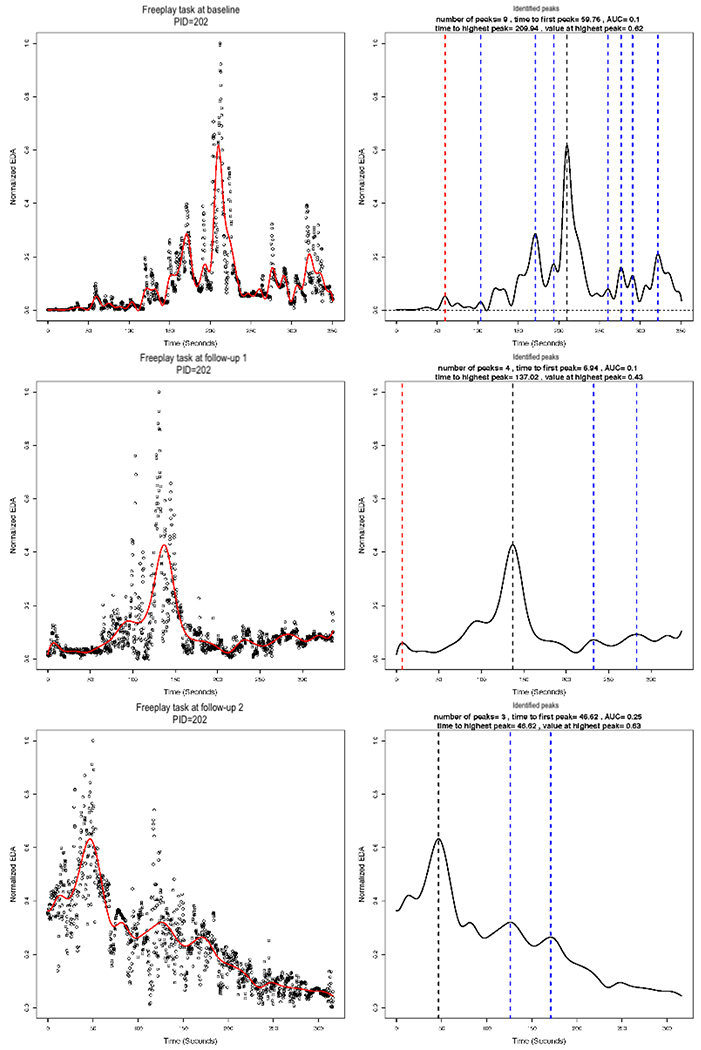
Electrodermal activity (EDA) for the free-play task at baseline, 3-month, and 6-month assessments for one mother (patient ID=202). The left column shows the estimated B-spline smoothed function overlaying the normalized EDA values. The right column shows the identified peaks and the values for peak features. The red dashed vertical lines demarcate the first peak, the black dashed vertical lines demarcate the highest peak, and the blue dashed vertical lines demarcate all other identified peaks. AUC: area under the curve; EDA: electrodermal activity; PID: patient ID.

**Figure 10.**
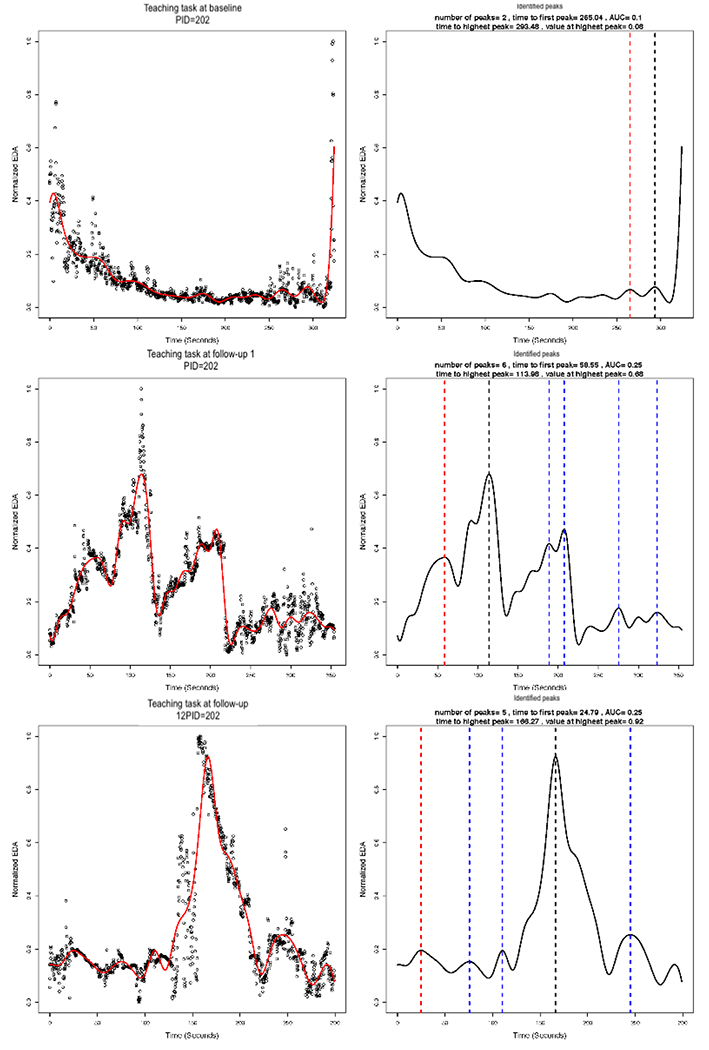
Electrodermal activity (EDA) for the teaching task at baseline, 3-month, and 6-month assessments for one mother (patient ID=202). The left column shows the estimated B-spline smoothed function overlaying the normalized EDA values. The right column shows the identified peaks and the values for peak features. The red dashed vertical lines demarcate the first peak, the black dashed vertical lines demarcate the highest peak, and the blue dashed vertical lines demarcate all other identified peaks AUC area under the curve; EDA: electrodermal activity; PID: patient ID.

In implementing the smoothing via B-splines, we used 13 basis functions. We plotted the smoothed versus normalized EDA for each AM, for each task, and for each assessment. The plots for the same AM illustrated in Figures 3 and 4 are shown in Figure 7, left column. The figure illustrates a potential problem with the B-spline approach, which relies on a cubic polynomial. To fit the first peak, the cubic function, by definition, must first increase and then decrease before increasing again. In so doing, the function imposes a false peak just before the actual first peak, and therefore, the function does not fit the actual data well (Figure 7). A different polynomial could be chosen for fitting the B-splines, but it would also impose a particular functional form that may not fit the actual data well. Figure 8 shows the EDA profiles for the teaching task for the same AM (PID 102), as shown in Figure 7. In comparison with the local linear regression approach illustrated in Figure 3, the B-spline approach illustrated in Figure 8 appears to fit at least as well for PID 102. In addition, the B-spline approach fits some individuals better than the local linear regression (not shown). For example, EDA for the free-play task for one intervention group AM is presented in Figure 9, and EDA for the teaching task for the same AM is presented in Figure 10. For this AM, the B-spline approach seemed to work well. We will discuss the 2 smoothing approaches further in the Discussion section.

### Computing Features of the Identified Peaks

Next, we identified peaks and computed various features of the peaks from the estimated EDA profiles. Figure 7 (right column) illustrates the identified peaks and presents the values for the peak features. The red dashed vertical lines demarcate the first peak, the black dashed vertical lines demarcate the highest peak, and the blue dashed vertical lines demarcate all other identified peaks. The time to first peak and the highest peak (both in seconds), the normalized EDA value at the highest peak (ie, amplitude), the drop from the highest peak (ie, reactivity), and the number of peaks are all presented in Figure 7, right column for one AM. The peaks were identified, and the features were computed for all AMs. These may be saved in a file for statistical analysis. Our sample size was ultimately too small for statistical significance testing between the intervention and control groups (ie, between-subjects), but we present descriptive statistics of the peak features in Table 1.

## Discussion

Our goal was to present 2 approaches for smoothing and summarizing EDA data by identifying EDA peaks and computing features of those peaks, including the number of peaks, time to first peak, time to highest peak, amplitude of the highest peak, and the time it takes to drop from the highest peak (ie, reactivity). These are certainly not all the features that could be constructed; rather, these are the ones that we thought may be most informative based on the laboratory EDA literature. It is quite possible that other features might be more interesting for ambulatory EDA. Similarly, the approaches we presented for smoothing the data are not the only 2 options [15,16]. Future research needs to examine other possible smoothing methods because the 2 approaches we used did not fit some individuals very well. On the other hand, there may be data for which these methods will fit better than others, as it really depends on the characteristics of a particular dataset. Even within our own data, we found instances where one approach fits the data better than the other and vice versa. Smoothing, identification, and construction of peak features are dependent on data quality.

A larger issue with regard to the use of EDA data for measuring stress, particularly in the ambulatory setting, is the degree to which it is an ecologically valid measure of stress. In the ambulatory setting, it is particularly difficult to distinguish emotional arousal from, for example, hand motions. It may be that the 2 of these are correlated to some degree because if people are emotionally aroused while talking, they may be moving their hands around a lot. Although the device contains an accelerometer, this does not entirely solve this problem. Other devices have been proposed for assessing stress that do not involve EDA. For example, the cStress device triangulates on stress by measuring respiration and heart rate variability via a chest strap [21]. On the other hand, a chest strap device is not as appealing as a wrist-worn device for most participants. Nevertheless, as the technology for the measurement of ambulatory EDA improves, the longitudinal measurement of EDA may have significant clinical implications, as providers and patients can review patterns of arousal that correspond to particular environmental stressors at specific times of day and help patients plan strategies for dealing with these stressors [22]. Similarly, the measurement of EDA may enable better stress management in the workplace, particularly in highly stressful occupations [23].

### Limitations

As mentioned previously, a lot of data were lost because the electrodes did not have a good connection with the skin, which resulted in EDA values that were approximately 0. Thus, the first lesson is to make sure that there is a good connection between the dry electrodes on the device and the skin. This means that the electrodes must be touching the skin on the ventral side of the wrist, which means that the device must be fairly tight on the wrist. People often do not wear watches, bands, or bracelets as tight as is necessary for the EDA device. Therefore, we suggest real-time monitoring of incoming EDA data so that if all the values are approximately 0, the participant could be contacted to inform them to tighten the device. Note that simply detecting that data are not being recorded in real time is not enough; there must be real-time detection of data that is being recorded but is essentially 0. Some of our EDA data were not usable because the band lost contact with the skin during some tasks, particularly early in the interview. By *loose connection*, we mean that the electrodes on the band did not have a good connection with the skin. We do not mean that the band was not connected in terms of recording the EDA. If the band was not recording, then EDA was missing at that particular measurement. This did happen sometimes but not enough to present a problem, particularly because we were able to smooth over it using the proposed smoothing approaches. It should be noted that the loose connection problem is not limited to the particular device, the iCalm, that we used. Empatica E4 also has this problem, as we have used it in a pilot study [24] and lost data because of a loose connection. Other studies using wrist-based ambulatory measures of EDA with the Affectiva Q Sensor have reported greater success [25,26] in both controlled and uncontrolled settings. However, other studies using the Affectiva Q sensor have reported data lost to electrode connection problems (eg, 31% [14] and 17% [22]). A total of 22.5% of data were lost because of electrode connection problems for the free-play task and 24% for the teaching task.

Owing to the loose connection problems during data collection, our sample size was substantially reduced, which limits our ability to draw conclusions about the between-subject effects of the intervention on the features constructed from the EDA data. Nevertheless, we think it is important to draw attention to data collection challenges as the measurement of ambulatory EDA has become more popular. In addition, researchers currently collecting EDA data have limited guidance on how to preprocess the data before statistical analysis.

A second challenge in EDA data collection is motion artifacts. These are identifiable as sharp spikes or drops in EDA data. One concern with the free-play task is that there were many more potential motion artifacts than there were in the teaching task because the AMs may have been moving around more during the free-play task. Another issue that may sometimes occur is that the EDA during the current task could be affected by the EDA response to the previous task. In our study, we tried to minimize this by having recovery periods between tasks, but, of course, there is no guarantee that the recovery periods were long enough. An individual’s EDA does not always return to the previous baseline, so it can be difficult to assess if the recovery period is long enough.

### Conclusions

In summary, in light of the increase in wearables for the continuous, ambulatory collection of EDA and associated challenges collecting data outside of the laboratory, analytic methods for computing various features of EDA are needed to help meet these challenges. We described 2 methods for smoothing and summarizing EDA data using FDA and local polynomial regression with AR errors. As the technology of wearable devices continues to advance, future research opportunities abound for improvement in device design that may improve the electrodes and their connection with the skin and facilitate in the development of improved methods for smoothing the data, identifying peaks, and constructing features.

## Supplementary Material

Appendix 1

## Figures and Tables

**Table 1.** Descriptive statistics for peak features by task among those adolescent mothers with identified peaks.

Peak feature/task	Baseline, mean (SD)	Follow-up^a^, mean (SD)
**Time to the highest peak (seconds)**		
Stroop	122.14 (63.42)	103.30 (61.45)
Teaching	144.29 (87.07)	123.55 (94.04)
Free play	201.79 (87.07)	215.10 (93.17)
**Value at highest peak (μS)**		
Stroop	0.67 (0.21)	0.75 (0.21)
Teaching	0.50 (0.29)	0.67 (0.22)
Free play	0.59 (0.26)	0.48 (0.25)
**Drop from the highest peak (seconds)**		
Stroop	13.18 (10.76)	17.91 (18.87)
Teaching	30.12 (17.49)	27.90 (43.01)
Free play	29.86 (24.71)	27.76 (18.41)
**Number of peaks**		
Stroop	6.39 (3.34)	5.13 (3.05)
Teaching	4.14 (3.51)	6.86 (3.61)
Free play	5.59 (2.95)	5.71 (3.33)
**Time to the first peak (seconds)**		
Stroop	45.66 (36.14)	51.49 (49.47)
Teaching	90.61 (86.08)	27.73 (18.91)
Free play	95.78 (69.93)	81.25 (90.94)

a Note that follow-up is at 3 months unless it was missing, in which case, the 6-month follow-up assessment for that individual was used (n=23 adolescent mothers [AMs] for the Stroop task, n=14 AMs for the teaching task at both baseline and follow-up, and n=17 AMs for the free-play task at both baseline and follow-up).

## Abbreviations

AMs: adolescent mothers
AR: autoregressive
AUC: area under the curve
EDA: electrodermal activity
FDA: functional data analysis
FIR: finite impulse response
GCV: generalized cross-validation
NIDA: National Institute on Drug Abuse
NIEHS: National Institute on Environmental Health Sciences
PI: principal investigator
PID: patient ID
PSP: Power Source Parenting
SC: skin conductance
SCL: skin conductance level
SCR: skin conductance response
SVM: support vector machine
TLP: transitional living program
