# Supplementary material for: Challenges and Opportunities in Collecting and Modeling Ambulatory Electrodermal Activity Data
Source: JMIR Biomed Eng. Author manuscript; Available in PMC 2021 Dec 8. (PMC8653913; doi:10.2196/17106)
Supplement: Appendix 1 [file NIHMS1750819-supplement-Appendix_1.docx]

Appendix

Functional Data Analysis Using B-Splines

library(fda)
library(plyr)
library(rootSolve)
library(pracma)

# function to select optimal lambda for the smoothing spline
GCV.lambda<-function(lower, upper, grid, time, response,K)
{
 loglam = seq(lower, upper, grid)
 Gcvsave = rep(NA, length(loglam))
 names(Gcvsave) = loglam
 Dfsave = Gcvsave
 basis <- create.bspline.basis(c(0, max(unique(time))),nbasis=K,norder=4)
 for(i in 1:length(loglam)){
 tempfdPari = fdPar(basis, Lfdobj=1, 10^loglam[i])
 Sm.i = smooth.basis(time,response,tempfdPari)
 Gcvsave[i] = sum(Sm.i$gcv)
 Dfsave[i] = Sm.i$df
 }
 optim.i<-which.min(Gcvsave)
 list(lambda=10^loglam[optim.i], min.gcv=Gcvsave[optim.i])
}

# function to select number of knots by method proposed by Ruppert(2000)
select.knots<-function(knots.seq, time, response, lower=-12, higher=12, grid=0.4)
{
 gcv<-rep(0,length(knots.seq))
 for(i in 1:(length(knots.seq)-1))
 {
 k1=knots.seq[i]
 k2=knots.seq[i+1]
 tempk1<-GCV.lambda(lower, higher, grid, time, response, k1)
 tempk2<-GCV.lambda(lower, higher, grid, time, response, k2)
 gcvk1=tempk1$min.gcv
 gcvk2=tempk2$min.gcv
 gcv[c(i,i+1)]=c(gcvk1,gcvk2)
 if(gcvk2 > 0.98*gcvk1)
 {
 optim.knots<-k2
 optim.lambda<-tempk2$lambda
 optim.gcv<-gcvk2
 break
 }
 }
 if(i==(length(knots.seq)-1)) {
 optim.knots<-k2
 optim.lambda<-tempk2$lambda
 optim.gcv<-gcvk2
 }
 list(knots=optim.knots, gcv=optim.gcv, lambda=optim.lambda)
}

# function to find value of fd at any value of x
valuefd<-function(x, basis, fd)
{
 basismat2 <- eval.basis(x,basis)
 value<-basismat2%*%fd$coef
 return(value)
}

# function to find value of the 1st derivatives of fd at any value of x
D1fd<-function(x,basis,fd)
{
 basismat2 <- eval.basis(x,basis,1)
 Dvalue<-basismat2%*%fd$coef
 return(Dvalue)
}

# function to find value of the 2nd derivatives of fd at any value of x
D2fd<-function(x,basis,fd)
{
 basismat2 <- eval.basis(x,basis,2)
 Dvalue<-basismat2%*%fd$coef
 return(Dvalue)
}

# function to identify useful peaks in the fitted curve
peak.filter<-function(ELAPSEDSECS,marker,root,basis,fd,threshold=0.1)
{
 drop=rep(0, length=length(marker))
 rightdrop=rep(0, length=length(marker))
 for(i in 1:length(marker))
 {

#if first local maximum is the first root value
 if((marker[i]==1)&(marker[i]!=length(root))) {
 if(((marker[i]+1) %in% marker)==FALSE)
 {
 if((valuefd(root[marker[i]],basis,fd)-valuefd(ELAPSEDSECS[1],basis,fd))>(valuefd(root[marker[i]],basis,fd)-valuefd(root[marker[i]+1],basis,fd)))
 {
 drop[i]=min(valuefd(root[marker[i]],basis,fd)-valuefd(ELAPSEDSECS[1],basis,fd),valuefd(root[marker[i]],basis,fd)-valuefd(root[marker[i]+1],basis,fd))
 rightdrop[i]=valuefd(root[marker[i]],basis,fd)-valuefd(root[marker[i]+1],basis,fd)
 } else {
 drop[i]=0
 rightdrop[i]=valuefd(root[marker[i]],basis,fd)-valuefd(root[marker[i]+1],basis,fd)
 }
 }
 } else if((marker[i]==1)&(marker[i]==length(root)))
 {
 drop[i]=min(valuefd(root[marker[i]],basis,fd)-valuefd(max(ELAPSEDSECS),basis,fd), valuefd(root[marker[i]],basis,fd)-valuefd(ELAPSEDSECS[1],basis,fd))
 rightdrop[i]=valuefd(root[marker[i]],basis,fd)-valuefd(max(ELAPSEDSECS),basis,fd)
 }else if((marker[i]!=1)&(marker[i]==length(root)))

#if last local maximum is the last root value
 {
 if(((marker[i]-1) %in% marker)==FALSE)
 {
 if((valuefd(root[marker[i]],basis,fd)-valuefd(max(ELAPSEDSECS),basis,fd))>(valuefd(root[marker[i]],basis,fd)-valuefd(root[marker[i]-1],basis,fd)))
 {
 drop[i]=min(valuefd(root[marker[i]],basis,fd)-valuefd(max(ELAPSEDSECS),basis,fd),valuefd(root[marker[i]],basis,fd)-valuefd(root[marker[i]-1],basis,fd))
 rightdrop[i]=valuefd(root[marker[i]],basis,fd)-valuefd(max(ELAPSEDSECS),basis,fd)
 } else {
 drop[i]=0
 rightdrop[i]=valuefd(root[marker[i]],basis,fd)-valuefd(max(ELAPSEDSECS),basis,fd)
 }
 }
 } else if((((marker[i]-1)%in%marker)==FALSE) & (((marker[i]+1)%in%marker)==FALSE))
 {
 drop[i]=min(valuefd(root[marker[i]],basis,fd)-valuefd(root[marker[i]-1],basis,fd), valuefd(root[marker[i]],basis,fd)-valuefd(root[marker[i]+1],basis,fd))
 rightdrop[i]=valuefd(root[marker[i]],basis,fd)-valuefd(root[marker[i]+1],basis,fd)
 }
 }
 list(drop=drop, rightdrop=rightdrop[drop>threshold], peaks=root[marker[drop>threshold]])
}

# function to prepare for the R plots and calculate features related to peaks
for.plot<-function(ELAPSEDSECS, EDA)
{
 knots.selec<-select.knots(seq(from=5, to=50, by=5), ELAPSEDSECS, EDA)
 K <- knots.selec$knots
 upper.time<-max(unique(ELAPSEDSECS))
 basis <- create.bspline.basis(c(0, upper.time),nbasis=K,norder=4)

 fdPar = fdPar(basis, 1, knots.selec$lambda)
 fdSmoothpenal = smooth.basis(ELAPSEDSECS, EDA, fdPar)
 fd= fdSmoothpenal$fd

 root=uniroot.all(function(x) D1fd(x,basis, fd),c(0,upper.time))

#index of root which are local maximum among all value with derivative zero.
 marker<-which(D2fd(root,basis,fd)<0)

local.max<-root[marker] #actual location of local maximum

 AUC<-trapz(seq(from=0, to=upper.time,length.out = length(ELAPSEDSECS)),predict(fdSmoothpenal))
 peaks<-peak.filter(ELAPSEDSECS,marker, root, basis,fd, 0.02)
 idt.peaks<-peaks$peaks
 right.drop<-peaks$rightdrop
 highest.peak.index<-which.max(valuefd(idt.peaks, basis, fd))
 highest.peak.drop<-peaks$rightdrop[highest.peak.index]

 list(fdSmoothpenal=fdSmoothpenal, peaks=idt.peaks,highest.peak.drop=highest.peak.drop, highest.peak.index=highest.peak.index, AUC=AUC, basis=basis, fd=fd)
}

### function to create graphs for given EDA observations.
### It produces two plots: one on original EDA plot with fitted curve;
### one on fitted curve marked with identified peaks and calculated feature### on peaks.
produce.graph<-function(ELAPSEDSECS, EDA, temp.obj, left.title)
{
 plot(ELAPSEDSECS, EDA, ylab="Normalized EDA", xlab="Time (Seconds)",ylim=c(0,1), main= left.title)
 lines(ELAPSEDSECS, predict(temp.obj$fdSmoothpenal),col=2,lwd=2)

 plot(temp.obj$fd, ylab="Normalized EDA", xlab="Time (Seconds)", ylim=c(0,1),lwd=2) # plot the derivatives
 title(main=paste("Identified Peaks",
 "\nnumber of peaks=",length(temp.obj$peaks), ",",
 "time to first peak=", round(temp.obj$peaks[1],2), ",",
 "AUC=",round(temp.obj$AUC/max(ELAPSEDSECS),2),
 "\ntime to highest peak=", round(temp.obj$peaks[temp.obj$highest.peak.index],2),",",
 "value at highest peak=", round(valuefd(temp.obj$peaks[temp.obj$highest.peak.index], temp.obj$basis, temp.obj$fd),2),",",
 "\ndrop of highest peak=", round(temp.obj$highest.peak.drop,2)))
 abline(v=temp.obj$peaks[1], col=2, lty=2,lwd=2)
 abline(v=temp.obj$peaks[temp.obj$highest.peak.index], col=1, lty=2,lwd=2)
 abline(v=temp.obj$peaks[-c(1,temp.obj$highest.peak.index)], col=4, lty=2,lwd=2)
}

################ Example code ####################
#setwd("desired_working_path")
PID.seq<-c(202)

### The following loop will run through all the PID in the PID.seq, and each ### run only consider one task
### This code will generate three pdf files for each PID, one for each task ### and named "tasknamePID.pdf".
### Each pdf file have two plots for each time period including baseline, ### followup1 and followup2:
### one on origianl EDA plot with fitted curve;
### one on fitted curve marked with identified peaks and features on peaks

for(k in PID.seq)
{
 #### load data for tasks at baseline
 #load("data_at_baseline")
 desired.PID=k
 stroopb <- all.dat[((all.dat$PID==desired.PID) & (!is.na(all.dat$STROOP)) & (all.dat$STROOP == "Stroop") & (all.dat$OBSTIME > mytime) & (!is.na(all.dat$EDA.normStroop))),]
 stroopb$ELAPSEDSECS <- unlist(lapply(split(stroopb, stroopb$PID), function(x){x$OBSTIME - x$OBSTIME[1]}))
 EDA.stroopb <- 1-stroopb$EDA.normStroop

 videob <- all.dat[((all.dat$PID==desired.PID) & (!is.na(all.dat$VIDEO)) & (all.dat$VIDEO == "Videotaping Teaching") & (all.dat$OBSTIME > mytime) & (!is.na(all.dat$EDA.normVideo))),]
 videob$ELAPSEDSECS <- unlist(lapply(split(videob, videob$PID), function(x){x$OBSTIME - x$OBSTIME[1]}))
 EDA.videob <- 1-videob$EDA.normVideo

 freeplayb <- all.dat[((all.dat$PID==desired.PID) & (!is.na(all.dat$freeplay)) & (all.dat$freeplay == "Free-play") & (all.dat$OBSTIME > mytime) & (!is.na(all.dat$EDA.normFreePlay))),]
 freeplayb$ELAPSEDSECS <- unlist(lapply(split(freeplayb, freeplayb$PID), function(x){x$OBSTIME - x$OBSTIME[1]}))
 EDA.freeplayb <- 1-freeplayb$EDA.normFreePlay

 #### load data for tasks at follow up 1
 #load("data_at_follow_up1")
 desired.PID=k
 stroopf1 <- all.dat[((all.dat$PID==desired.PID) & (!is.na(all.dat$STROOP)) & (all.dat$STROOP == "Stroop") & (all.dat$OBSTIME > mytime) & (!is.na(all.dat$EDA.normStroop))),]
 stroopf1$ELAPSEDSECS <- unlist(lapply(split(stroopf1, stroopf1$PID), function(x){x$OBSTIME - x$OBSTIME[1]}))
 EDA.stroopf1 <- 1-stroopf1$EDA.normStroop

 videof1 <- all.dat[((all.dat$PID==desired.PID) & (!is.na(all.dat$VIDEO)) & (all.dat$VIDEO == "Videotaping Teaching") & (all.dat$OBSTIME > mytime) & (!is.na(all.dat$EDA.normVideo))),]
 videof1$ELAPSEDSECS <- unlist(lapply(split(videof1, videof1$PID), function(x){x$OBSTIME - x$OBSTIME[1]}))
 EDA.videof1 <- 1-videof1$EDA.normVideo

 freeplayf1 <- all.dat[((all.dat$PID==desired.PID) & (!is.na(all.dat$freeplay)) & (all.dat$freeplay == "Free-play") & (all.dat$OBSTIME > mytime) & (!is.na(all.dat$EDA.normFreePlay))),]
 freeplayf1$ELAPSEDSECS <- unlist(lapply(split(freeplayf1, freeplayf1$PID), function(x){x$OBSTIME - x$OBSTIME[1]}))
 EDA.freeplayf1 <- 1-freeplayf1$EDA.normFreePlay

 #### load data for tasks at follow up 2
 #load("data_at_follow_up2")
 desired.PID=k
 stroopf2 <- all.dat[((all.dat$PID==desired.PID) & (!is.na(all.dat$STROOP)) & (all.dat$STROOP == "Stroop") & (all.dat$OBSTIME > mytime) & (!is.na(all.dat$EDA.normStroop))),]
 stroopf2$ELAPSEDSECS <- unlist(lapply(split(stroopf2, stroopf2$PID), function(x){x$OBSTIME - x$OBSTIME[1]}))
 EDA.stroopf2 <- 1-stroopf2$EDA.normStroop

 videof2 <- all.dat[((all.dat$PID==desired.PID) & (!is.na(all.dat$VIDEO)) & (all.dat$VIDEO == "Videotaping Teaching") & (all.dat$OBSTIME > mytime) & (!is.na(all.dat$EDA.normVideo))),]
 videof2$ELAPSEDSECS <- unlist(lapply(split(videof2, videof2$PID), function(x){x$OBSTIME - x$OBSTIME[1]}))
 EDA.videof2 <- 1-videof2$EDA.normVideo

 freeplayf2 <- all.dat[((all.dat$PID==desired.PID) & (!is.na(all.dat$freeplay)) & (all.dat$freeplay == "Free-play") & (all.dat$OBSTIME > mytime) & (!is.na(all.dat$EDA.normFreePlay))),]
 freeplayf2$ELAPSEDSECS <- unlist(lapply(split(freeplayf2, freeplayf2$PID), function(x){x$OBSTIME - x$OBSTIME[1]}))
 EDA.freeplayf2 <- 1-freeplayf2$EDA.normFreePlay

 ### Prepare data for generating plots
 stroopb.res<-for.plot(stroopb$ELAPSEDSECS, EDA.stroopb)
 stroopf1.res<-for.plot(stroopf1$ELAPSEDSECS, EDA.stroopf1)
 stroopf2.res<-for.plot(stroopf2$ELAPSEDSECS, EDA.stroopf2)

 videob.res<-for.plot(videob$ELAPSEDSECS, EDA.videob)
 videof1.res<-for.plot(videof1$ELAPSEDSECS, EDA.videof1)
 videof2.res<-for.plot(videof2$ELAPSEDSECS, EDA.videof2)

 freeplayb.res<-for.plot(freeplayb$ELAPSEDSECS, EDA.freeplayb)
 freeplayf1.res<-for.plot(freeplayf1$ELAPSEDSECS, EDA.freeplayf1)
 freeplayf2.res<-for.plot(freeplayf2$ELAPSEDSECS, EDA.freeplayf2)

 ### produce graphs for each task-PID combination
 outfile <- sprintf("stroop%i.pdf", k)
 pdf(outfile,width=14, height=7)
 par(mar=c(5.1,4.1,6.1,2.1))
 par(mfrow=c(1,2), cex.lab=1.4, cex.main=1.4)
 produce.graph(stroopb$ELAPSEDSECS, EDA.stroopb, stroopb.res, paste("Stroop Task at Baseline \nPID=",k))
 produce.graph(stroopf1$ELAPSEDSECS, EDA.stroopf1, stroopf1.res, paste("Stroop Task at Follow Up 1 \nPID=",k))
 produce.graph(stroopf2$ELAPSEDSECS, EDA.stroopf2, stroopf2.res, paste("Stroop Task at Follow Up 2 \nPID=",k))
 dev.off()

 outfile <- sprintf("video%i.pdf", k)
 pdf(outfile,width=14, height=21)
 par(mar=c(5.1,4.1,6.1,2.1))
 par(mfrow=c(3,2), cex.lab=1.4, cex.main=1.4)
 produce.graph(videob$ELAPSEDSECS, EDA.videob, videob.res, paste("Teaching Task at Baseline \nPID=",k))
 produce.graph(videof1$ELAPSEDSECS, EDA.videof1, videof1.res, paste("Teaching Task at Follow Up 1 \nPID=",k))
 produce.graph(videof2$ELAPSEDSECS, EDA.videof2, videof2.res, paste("Teaching Task at Follow Up 2 \nPID=",k))
 dev.off()

 outfile <- sprintf("freeplay%i.pdf", k)
 pdf(outfile,width=14, height=21)
 par(mar=c(5.1,4.1,6.1,2.1))
 par(mfrow=c(3,2), cex.lab=1.4, cex.main=1.4)
 produce.graph(freeplayb$ELAPSEDSECS, EDA.freeplayb, freeplayb.res, paste("Freeplay Task at Baseline \nPID=",k))
 produce.graph(freeplayf1$ELAPSEDSECS, EDA.freeplayf1, freeplayf1.res, paste("Freeplay Task at Follow Up 1 \nPID=",k))
 produce.graph(freeplayf2$ELAPSEDSECS, EDA.freeplayf2, freeplayf2.res, paste("Freeplay Task at Follow Up 2 \nPID=",k))
 dev.off()
}

Local Linear Regression with AR errors

### implementation of local linear regression for data with AR errors ####
library(locpol)
library(locfit)
library(rqPen)
library(KernSmooth)

#####################################################################
# Some functions to be used by the main function llwar #
#####################################################################

#### 1. matrix power ####
matpower <- function(a,alpha){
 small <- .00000001
 p1<-nrow(a)
 eva<-eigen(a)$values
 eve<-eigen(a)$vectors
 eve<-eve/t(matrix((diag(t(eve)%*%eve)^0.5),p1,p1))
 index<-(1:p1)[abs(eva)>small]
 evai<-eva
 evai[index]<-(eva[index])^(alpha)
 ai<-eve%*%diag(evai,length(evai))%*%t(eve)
 return(ai)
}
#### 2.compute kernel term #####
kern <- function(x,h,i){
 if(is.matrix(x)==TRUE) {
 del <- t(t(x)-x[i,])
 r<-ncol(x)} else {
 del <- x-x[i]
 r<-1}
 ndel <- diag(del%*%t(del))
 w <- ((1/sqrt(2*pi))^r)*(1/h^r)*exp((-1/2)*ndel/h^2)
 w <- c(w)
 w1 <- w/sum(w)
 return(w1)
}

#### 3. local linear regression smoother #####
lls.term <- function(x,w){
 b <- matpower(t(x*w)%*%(x),-1)%*%(t(x*w))
 return(b)
}

lls <- function(x,h){
 n <- length(x)
 ab <- numeric(0)

 for(i in 1:n){
 del <- cbind(1,t(t(x) - x[i]))
 w <- kern(x,h,i)
 abi <- lls.term(del,w)[1,]
 ab <- rbind(ab,abi)
 }
 return(ab)
}

###########################################################################
# Main function llwar to fit the local linear regression with #
# AR errors (Li and Li 2006) on observations (x, y) #
###########################################################################
## input: x= time sequence of observation,
## y= observed (normalized) EDA values for
## d= initial number of terms in the AR model for the error, will be s## elected by penalization method. Default value is set to be 15.

## output: hat.m= fitted value of y
llwar<-function(x, y, d=15)
{
 df=data.frame(x=x,y=y)
 n=length(x)

 # under working independence, fit the model then calculate the residuals
 bw_1 <- pluginBw(df$x, df$y, deg=1, kernel=gaussK)
 m_I <- locLinSmootherC(df$x, df$y, x, bw_1, kernel=gaussK)
 pred_y1<-m_I$beta0
 epsilon<-y-pred_y1

 #creating independent errors e
 E=matrix(0, ncol=d, nrow=n-d)
 for(i in 1:(n-d))
 {
 e_t=epsilon[c((i+d-1):i)]
 E[i,]=e_t
 }

 # find estimates by the difference based method
 dbe.y=diff(y[-c(1:d)])
 dbe.x=diff(x[-c(1:d)])
 dbe.e=apply(E,2,diff)
 beta.dbe<-lm(dbe.y~dbe.e)$coef[-1]

 y_star<-y[-c(1:d)]-E%*%(beta.dbe)
 bw_2 <- thumbBw(df$x[-c(1:d)], y_star, deg=1, kernel=gaussK)

 local.linear.smoother=lls(x,bw_2)
 S_h=local.linear.smoother[(d+1):n,(d+1):n] #find the local linear smoother

 I_S=(diag(1,n-d)-S_h) # I_n-S_h
 EI_S=(I_S)%*%E #E(I_n-S_h)

 #find the unpenalized estimated coefficients
 hat.beta<-matpower(t(EI_S)%*%(EI_S),-1)%*%t(EI_S)%*%I_S%*%y[-c(1:d)]
 hat.m<-S_h%*%(y[-c(1:d)]-E%*%hat.beta)
 hat.eta<-y[-c(1:d)]-E%*%hat.beta-hat.m
 hat.sigma<-sd(hat.eta)

 F=E
 temp=matrix(0,d,d)
 for(i in 1:(n-d))
 {
 temp<-temp+(F[i,])%*%t(F[i,])
 }
 se.beta.ls<-hat.sigma*sqrt(diag(matpower(temp,-1)))

 lambda.seq<-seq(from=0.1/sqrt(n), to=2*sqrt(log(n))/sqrt(n), length.out=20)
 BIC<-rep(0,length(lambda.seq))
 BIC.optimal=1000
 for(j in 1:length(lambda.seq))
 {
 lambda.constant<-lambda.seq[j]
 lambda<-lambda.constant*se.beta.ls
 beta.new<-hat.beta
 iteration=1
 criterion=1
 beta.opt=NULL

 while(criterion>1e-4)
 {
 beta.temp<-beta.new
 S.diagonal<-NULL
 for(i in 1:d)
 {
 S.diagonal<-c(S.diagonal, scad_deriv(beta.temp[i],lambda[i],3.7)/abs(beta.temp)[i])
 }
 Sigma.mat<-diag(S.diagonal)
 beta.new<-matpower(t(EI_S)%*%(EI_S)+n*Sigma.mat, -1)%*%t(EI_S)%*%I_S%*%y[-c(1:d)]
 criterion<-sum((beta.temp-beta.new)^2)
 iteration<-iteration+1
 }
 beta.non0.index<-I(abs(beta.new)>1e-10)
 beta.new<-beta.new*beta.non0.index

 D_tilde<- (t(EI_S)%*%(EI_S))[beta.non0.index, beta.non0.index]
 e_lambda<-sum(diag(matpower(D_tilde+Sigma.mat[beta.non0.index, beta.non0.index], -1)%*%D_tilde))

 RSS<-sum((I_S%*%y[-c(1:d)]-I_S%*%E%*%beta.new)^2)
 BIC.temp<-log(RSS/n)+e_lambda*log(n)/n
 BIC[j]=BIC.temp
 if(BIC.temp<BIC.optimal)
 {
 beta.opt<-beta.new
 }
 }

 final.hat.beta<-beta.opt
 hat.m<-S_h%*%(y[-c(1:d)]-E%*%final.hat.beta)
 return(hat.m)
}

# Function to produce graph with original observations and the fitted curves.
produce.graph<-function(ELAPSEDSECS, EDA, fitted.EDA, title)
{
 plot(ELAPSEDSECS, EDA, ylab="Normalized EDA", xlab="Time (Seconds)",ylim=c(0,1), main= title)
 lines(ELAPSEDSECS, (fitted.EDA),col=2,lwd=2)
}


###########################################################################
# Example: code to create plot for PID 202 #
###########################################################################

#setwd("desired-working-directory")
PID.seq<-c(202)

for(k in PID.seq)
{
 #### load data for all tasks at baseline
 #load("data_at_baseline")
 desired.PID=k
 stroopb <- all.dat[((all.dat$PID==desired.PID) & (!is.na(all.dat$STROOP)) & (all.dat$STROOP == "Stroop") & (all.dat$OBSTIME > mytime) & (!is.na(all.dat$EDA.normStroop))),]
 stroopb$ELAPSEDSECS <- unlist(lapply(split(stroopb, stroopb$PID), function(x){x$OBSTIME - x$OBSTIME[1]}))
 EDA.stroopb <- 1-stroopb$EDA.normStroop

 videob <- all.dat[((all.dat$PID==desired.PID) & (!is.na(all.dat$VIDEO)) & (all.dat$VIDEO == "Videotaping Teaching") & (all.dat$OBSTIME > mytime) & (!is.na(all.dat$EDA.normVideo))),]
 videob$ELAPSEDSECS <- unlist(lapply(split(videob, videob$PID), function(x){x$OBSTIME - x$OBSTIME[1]}))
 EDA.videob <- 1-videob$EDA.normVideo

 freeplayb <- all.dat[((all.dat$PID==desired.PID) & (!is.na(all.dat$freeplay)) & (all.dat$freeplay == "Free-play") & (all.dat$OBSTIME > mytime) & (!is.na(all.dat$EDA.normFreePlay))),]
 freeplayb$ELAPSEDSECS <- unlist(lapply(split(freeplayb, freeplayb$PID), function(x){x$OBSTIME - x$OBSTIME[1]}))
 EDA.freeplayb <- 1-freeplayb$EDA.normFreePlay

 #### load data for tasks at follow up 1
 #load("data_at_followup1")
 desired.PID=k
 stroopf1 <- all.dat[((all.dat$PID==desired.PID) & (!is.na(all.dat$STROOP)) & (all.dat$STROOP == "Stroop") & (all.dat$OBSTIME > mytime) & (!is.na(all.dat$EDA.normStroop))),]
 stroopf1$ELAPSEDSECS <- unlist(lapply(split(stroopf1, stroopf1$PID), function(x){x$OBSTIME - x$OBSTIME[1]}))
 EDA.stroopf1 <- 1-stroopf1$EDA.normStroop

 videof1 <- all.dat[((all.dat$PID==desired.PID) & (!is.na(all.dat$VIDEO)) & (all.dat$VIDEO == "Videotaping Teaching") & (all.dat$OBSTIME > mytime) & (!is.na(all.dat$EDA.normVideo))),]
 videof1$ELAPSEDSECS <- unlist(lapply(split(videof1, videof1$PID), function(x){x$OBSTIME - x$OBSTIME[1]}))
 EDA.videof1 <- 1-videof1$EDA.normVideo

 freeplayf1 <- all.dat[((all.dat$PID==desired.PID) & (!is.na(all.dat$freeplay)) & (all.dat$freeplay == "Free-play") & (all.dat$OBSTIME > mytime) & (!is.na(all.dat$EDA.normFreePlay))),]
 freeplayf1$ELAPSEDSECS <- unlist(lapply(split(freeplayf1, freeplayf1$PID), function(x){x$OBSTIME - x$OBSTIME[1]}))
 EDA.freeplayf1 <- 1-freeplayf1$EDA.normFreePlay

 #### load data for all tasks at follow up 2
 #load("data_at_followup2")
 desired.PID=k
 stroopf2 <- all.dat[((all.dat$PID==desired.PID) & (!is.na(all.dat$STROOP)) & (all.dat$STROOP == "Stroop") & (all.dat$OBSTIME > mytime) & (!is.na(all.dat$EDA.normStroop))),]
 stroopf2$ELAPSEDSECS <- unlist(lapply(split(stroopf2, stroopf2$PID), function(x){x$OBSTIME - x$OBSTIME[1]}))
 EDA.stroopf2 <- 1-stroopf2$EDA.normStroop

 videof2 <- all.dat[((all.dat$PID==desired.PID) & (!is.na(all.dat$VIDEO)) & (all.dat$VIDEO == "Videotaping Teaching") & (all.dat$OBSTIME > mytime) & (!is.na(all.dat$EDA.normVideo))),]
 videof2$ELAPSEDSECS <- unlist(lapply(split(videof2, videof2$PID), function(x){x$OBSTIME - x$OBSTIME[1]}))
 EDA.videof2 <- 1-videof2$EDA.normVideo

 freeplayf2 <- all.dat[((all.dat$PID==desired.PID) & (!is.na(all.dat$freeplay)) & (all.dat$freeplay == "Free-play") & (all.dat$OBSTIME > mytime) & (!is.na(all.dat$EDA.normFreePlay))),]
 freeplayf2$ELAPSEDSECS <- unlist(lapply(split(freeplayf2, freeplayf2$PID), function(x){x$OBSTIME - x$OBSTIME[1]}))
 EDA.freeplayf2 <- 1-freeplayf2$EDA.normFreePlay

 #Fit the loaded data by local linear regression with AR errors at baseline, #followup1 and followup2
 d=15
 stroopb.fit<-llwar(x=stroopb$ELAPSEDSECS, y=EDA.stroopb, d)
 stroopf1.fit<-llwar(x=stroopf1$ELAPSEDSECS, y=EDA.stroopf1, d)
 stroopf2.fit<-llwar(x=stroopf2$ELAPSEDSECS, y=EDA.stroopf2, d)

 videob.fit<-llwar(x=videob$ELAPSEDSECS, y=EDA.videob, d)
 videof1.fit<-llwar(x=videof1$ELAPSEDSECS, y=EDA.videof1, d)
 videof2.fit<-llwar(x=videof2$ELAPSEDSECS, y=EDA.videof2, d)

 freeplayb.fit<-llwar(x=freeplayb$ELAPSEDSECS, y=EDA.freeplayb, d)
 freeplayf1.fit<-llwar(x=freeplayf1$ELAPSEDSECS, y=EDA.freeplayf1, d)
 freeplayf2.fit<-llwar(x=freeplayf2$ELAPSEDSECS, y=EDA.freeplayf2, d)

 #create plots for raw data and its fit at baseline, followup1 and followup2
 outfile <- sprintf("stroop%i.pdf", k)
 pdf(outfile,width=15, height=5)
 par(mfrow=c(1,3), cex.lab=1.4, cex.main=1.4)
 produce.graph(stroopb$ELAPSEDSECS[-c(1:d)], EDA.stroopb[-c(1:d)], stroopb.fit, paste("Stroop Task at Baseline \nPID=",k))
 produce.graph(stroopf1$ELAPSEDSECS[-c(1:d)], EDA.stroopf1[-c(1:d)], stroopf1.fit, paste("Stroop Task at Follow Up 1 \nPID=",k))
 produce.graph(stroopf2$ELAPSEDSECS[-c(1:d)], EDA.stroopf2[-c(1:d)], stroopf2.fit, paste("Stroop Task at Follow Up 2 \nPID=",k))
 dev.off()

 outfile <- sprintf("video%i.pdf", k)
 pdf(outfile,width=15, height=5)
 par(mfrow=c(1,3), cex.lab=1.4, cex.main=1.4)
 produce.graph(videob$ELAPSEDSECS[-c(1:d)], EDA.videob[-c(1:d)], videob.fit, paste("Teaching Task at Baseline \nPID=",k))
 produce.graph(videof1$ELAPSEDSECS[-c(1:d)], EDA.videof1[-c(1:d)], videof1.fit, paste("Teaching Task at Follow Up 1 \nPID=",k))
 produce.graph(videof2$ELAPSEDSECS[-c(1:d)], EDA.videof2[-c(1:d)], videof2.fit, paste("Teaching Task at Follow Up 2 \nPID=",k))
 dev.off()

 outfile <- sprintf("freeplay%i.pdf", k)
 pdf(outfile,width=15, height=5)
 par(mfrow=c(1,3), cex.lab=1.4, cex.main=1.4)
 produce.graph(freeplayb$ELAPSEDSECS[-c(1:d)], EDA.freeplayb[-c(1:d)], freeplayb.fit, paste("Freeplay Task at Baseline \nPID=",k))
 produce.graph(freeplayf1$ELAPSEDSECS[-c(1:d)], EDA.freeplayf1[-c(1:d)], freeplayf1.fit, paste("Freeplay Task at Follow Up 1 \nPID=",k))
 produce.graph(freeplayf2$ELAPSEDSECS[-c(1:d)], EDA.freeplayf2[-c(1:d)], freeplayf2.fit, paste("Freeplay Task at Follow Up 2 \nPID=",k))
 dev.off()
}

Low-pass filter

library(signal)
## function to apply a lowpass.filter on
lowpass.filter<-function(input)
{
 filter.coef<-fir1(16, 1/length(input), "low")
 input.filter.fir<-rep(0,length(input))
 input.filter.fir[1:17]=input[1:17]
 for(i in 18:length(input))
 {
 input.filter.fir[i]=filter.coef%*%input[(i-17):(i-1)]
 }
 return(input.filter.fir)
}

#### Example code ####
EDA.filtered=lowpass.filter(EDA)
